# Oral Cancer: Population Sample of the State of Alagoas at a Reference Hospital

**DOI:** 10.1016/S1808-8694(15)30491-2

**Published:** 2015-10-19

**Authors:** Luiz Carlos Oliveira dos Santos, Maria Cristina Teixeira Cangussu, Olívio de Medeiros Batista, Jadileide Pereira dos Santos

**Affiliations:** 1 Doctoral student in stomatology, Integrated Graduate Dentistry Program (UFPB/UFBA). Adjunct professor of stomatology at UFAL; 2 Doctorate, adjunct professor of the Social and Pediatric Dentistry Department, FO-UFBA; 3 Doctorate, professor of stomatology at UFPB; 4 Specialist in health surveillance, social worker

**Keywords:** oral cavity, epidemiology, mouth neoplasms

## Abstract

The incidence and mortality of oral cancer in Brazil remains high; the disease manifests varying features throughout the country.

**Aim:**

To analyze the epidemiology of oral cancer, including the prevalence, type and site of lesions, the distribution in the state of Alagoas, staging, treatment, and social and demographic aspects.

**Materials and Methods:**

A descriptive retrospective study was carried out. Data were gathered from records of a hospital in Alagoas from January 2000 to December 2006.

**Results:**

Of 396 cases (100% of the sample), 62.70% were male and 37.30% female. Most tumors were on the tongue. The mean age was 61.95 years (SD=14.56 years), and 95.2% of the sample were aged over 40 years. Most of lesions were stage II (57.04%). Radiotherapy was the most common treatment.

**Conclusion:**

This study revealed the epidemiology of oral cancer patients at a reference hospital in the state of Alagoas. The results showed that oral cancer occurs mostly in males in the sixth decade of life; the most common site is the tongue, followed by the floor of the mouth.

## INTRODUCTION

Mortality due to chronic and degenerative diseases is increasing in Brazil; among these, malignant neoplasms are the second cause of death when external causes are excluded. Cancer, one of these chronic/degenerative diseases, negatively affects the biopsychosocial factors of an individual's life.^1^

Mouth cancer is a public health issue, since the incidence and mortality rates in this site are high among the population.^1^

The diagnosis in most patients with mouth cancers is generally made at advanced stages, possibly because such lesions are asymptomatic and apparently harmless at initial stages and thus are not validated by individuals or healthcare professionals.^2^,^3^ This suggests also that access to and the qualify of healthcare is poor.

The most important known risk factors for this disease are smoking and alcohol consumption; these habits are synergic in tumor development. Other factors, such as chronic mechanical irritation (poorly adapted dental appliances, fractures, and absence of teeth), chemical irritants (use of mouthwashes), and poor mouth hygiene, have also been incriminated as possible risk factors for mouth cancer.^4^,^5^

According to statistics of the Instituto Nacional de Cancer (National Cancer Institute, or INCA), mouth cancers are the 5th most common tumor in males and the 8th most common tumor in females.1 Its occurrence is highest in developing countries,^6^ which has been attributed to: poor mouth hygiene and use of maladapted dental appliances. Lip cancer is relevant in Brazil, which is a tropical country; its economy includes rural activities in which workers are exposed to sunlight.^1^, ^4^, ^5^

In the state of Alagoas, the INCA estimated that there would be 2,990 new cases in 2005; this is the only available data for this state, since there is no systematic registration with consistency assessments of this disease in the five hospitals classified as high complexity centers in oncology. Furthermore, the literature on the incidence and mortality due to cancer in this sate is sparse; this situation underlines the need for epidemiological studies of this state to enable planning of prevention, control and treatment measures for cancer.^7^

Thus, the purpose of this paper was to evaluate the epidemiological profile of mouth cancer, comprising the prevalence, type and site of tumors in patients seen at a hospital in the state of Alagoas from 2000 to2006. Data included the education level, tumor staging, treatment at the institution, and the social and demographic profile of patients, to generate data for healthcare units.

## MATERIAL AND METHOD

A retrospective study was made of 396 files of patients diagnosed with mouth squamous cell carcinomas, seen at a hospital in the state of Alagoas from 2000 to2006.

The institution is a reference hospital for cancer patients of the entire state of Alagoas; up to 2006 it was the only healthcare institution providing radiotherapy. A survey was made of the registration book of the pathology unit in the Maceio hospital from 2000 to 2006; patients registered as bringing specimens for histopathological analysis, and diagnosed with squamous cell carcinoma, were selected.

The following data were selected for analysis: age range, origin, gender, ethnic group, education level, occupation, life habits (drinking alcohol and smoking), site of primary tumors in the mouth, TNM staging of patients, and treatment.

The age prevalence was grouped into decades from 40 to 70 years; patients aged below or over this group were gathered into two separate groups. The distribution of cities of origin of patients was made according to the demographic density of geographic macro- and micro-regions of Alagoas as classified by the Brazilian Geographical and Statistical Institute (Instituto Brasileiro de Geografia e Estatística or IBGE).

Patients were also divided into male and female groups. Ethnic groups were those defined by the IBGE (white, brown, black, yellow and native Indians).

The parameter for defining the education level was based on the Education Law - LDB number 9.394/96.

The occupation was classified according to the relative distribution vis a vis the constant frequency in the files.

Patients who consumed alcoholic beverages were classified according to the duration of use and daily quantities ingested, regardless of the drink. Smokers were patients who smoked daily, regardless of the quantity, quality of tobacco, and type of cigarettes.

Primary tumors were classified according to the International Classification of Diseases - Oncology (ICD-O), which is used at the institution and is frequent in the files that were evaluated. Lesions were grouped according to anatomical sites, as follows: floor of the mouth, gingiva, lip, tongue, jugal mucosa, palate, and retromolar area.

Staging was based on the physical examination; tumors were classified according to the ICD-O,8 and the 1998 TNM classification of the International Union Against Cancer (UICC). Tumor stages were stage I-II (T1 or T2 and N0) and stage III-IV (T3, T4 or N>0).

The type of treatment was that described in the files; this included chemotherapy, radiotherapy, surgery, and other therapies.

Following data gathering, a descriptive analysis was made of absolute and relative frequencies for the categorical variables, central tendency measures, and dispersion of continuous variables. The Mantel-Haenzel chi-square test was applied for defining the differences among groups. The significance level was 5%.

The Research Ethics Committee of the institution approved this study (protocol number 005567/2006-17.

## RESULTS

There were 396 files during the study period. Some did not contain all data, or were filled in incorrectly; these were discarded during analysis.

The incidence was higher at the sixth decade of life. The mean age of patients was 61.95 years (SD=14.56), ranging from 26 years to 100 years. Males were more affected than females in all age groups; this difference was statistically significant in the fourth and the fifth decades of life. Females predominated in the 60+ years age group, (p<0.01) (Table 1).

**Table 1:** Distribution of mouth squamous cell carcinoma patients according to sex and age Maceio-AL, 2000-2006.

Age Range (in years)	Male		Female		Total	
	No	%	No	%	No	%
< 40	12	4,8	7	4,8	19	4,8
40 – 50	54	21,6*	15	10,3*	69	17,4
50 – 60	61	24,4*	18	12,3*	79	19,95
60 – 70	60	24,0	39	26,7	99	25,0
> 70	63	25,2*	67	45,9*	130	32,8
Total	250	100	146	100	396	100

* p<0,01

Most of the patients were rural workers (53.9%), followed by industry workers (3.2%), trade and independent professionals (3.2%).

The most frequent ethnic group was brown (69.19%), followed by white (27.02%); there were few black patients (3.28%).

The education level was low in the sample population; 26.55% were illiterate. Most patients had incomplete basic education (46.56%), full basic education (20.36%) and middle or higher education (9.54%).

The frequency was high on the tongue in both sexes; a higher prevalence in males was statistically significant (p=0.05). Squamous cell carcinoma was more frequent in the lip and soft palate in females compared to males (Table 2).

**Table 2:** Distribution of mouth squamous cell carcinoma patients according to the anatomical site Maceio-AL, 2000-2006.

Anatomical site	Male		Female		Total	
	No	%	No	%	No	%
Floor of mouth	47	18,87	26	17,69	73	18,43
Gingiva	8	3,21	3	2,04	11	2,78
Lip	16	6,43	23	15,65	39	9,85
Tongue	123	49,40*	58	39,46*	181	45,71
Jugal mucosa	22	8,83	7	4,76	29	7,32
Hard palate	18	7,23	18	12,24	36	9,10
Soft palate	10	4,02	11	7,48	21	5,30
Retromolar area	5	2,01	1	0,68	6	1,51
Total	249	100	147	100	396	100

* p=0,05

Most of the patients (57.04%) presented initially with stage II tumors; only 6 patients manifested incipient lesions (stage I). There were no gender differences. Staging information was found in 291 files; it was lacking in 105 files, which were discarded from the analysis (Table 3).

**Table 3:** Distribution of mouth squamous cell carcinoma patients according to staging of the lesion Maceio-AL, 2000-2006.

Staging of lesions	Male		Female		Total	
	No	%	No	%	No	%
Stage 1	2	1,11	4	3,60	6	2,06
Stage II	101	56,11	65	58,56	166	57,04
Stage III	52	28,89	31	27,93	83	28,52
Stage IV	25	13,89	11	9,91	36	12,37
Total	180	100	111	100	291	100

Radiotherapy was indicated in 47.68% of cases, more often in female patients (p<0.01); surgery was indicated next (24.74% of cases). Eight files did not bring this information, and were discarded from the analysis (Table 4).

**Table 4:** Distribution of mouth squamous cell carcinoma patients according to the type of treatment Maceio-AL, 2000-2006.

Type of treatment	Male		Female		Total	
	No	%	No	%	No	%
Radiotherapy	110	44,90*	75	52,45*	185	47,68
Surgery	60	24,49	36	25,17	96	24,74
Chemotherapy	5	2,04	1	0,70	6	1,55
Surgery + radiotherapy	45	18,37	23	16,08	68	17,53
Surgery + chemotherapy	5	2,04	3	2,10	8	2,06
Surgery + chemotherapy + radiotherapy	5	2,04	2	1,40	7	1,80
Radiotherapy + chemotherapy	15	6,12	3	2,10	18	4,64
Total	245	100	143	100	388	100

* p<0,01

Information about smoking and drinking alcoholic beverages was absent in many files - notifying these data is not compulsory. There were 36 files (9.09%) with data on smoking and 34 files (8.60%) with data on drinking alcohol.

Alagoas state has 102 municipalities; the highest frequency of mouth cancer was in the capital, Maceio (158 patients – 40.20%), followed by Arapiraca (29 patients – 7.38%). Other patients came from another 66 municipalities of the state, 67% of the total.

## DISCUSSION

The proportion of mouth cancer has increased year after year worldwide and in Brazil.^9^ This disease is being considered a public health issue because of high incidence and mortality rates.

The state of Alagoas has one of the worst human development indices (HDI) in Brazil; 10 this index assesses parameters such as education, life expectancy at birth, health conditions, per capita income (GDP), among others. In Alagoas, the HDI is 0.538, higher only than the state of Piaui.

Of 102 municipalities in Alagoas, 68 (67%) were represented in our sample; patients seek healthcare in the capital city (Maceio), showing the importance of care in this city for the state's population and the lack of decentralized medium and high complexity healthcare. Municipalities absent from these numbers may reflect geographical distance and precarious roads from rural areas, as well as lack of effective referencing and counter-referencing between the basic health system and cancer reference hospitals.

Factors affecting population health are closely related with economic issues and federal public healthcare policies. The population of Alagoas is 2,843,278 people; there are currently 6,136 hospital beds in this state (2.16 beds per 1,000 inhabitants), which is below the recommended number by the Ministry of Health (2.5 to 3-0 beds per 1,000 people).

There are only five high complexity centers in oncology accredited by the Brazilian Unified Health System (SUS), which are not enough for the demand. These centers are distributed as follows: one in the rural area and four in the state capital (Maceio)^11^ where until mid-2007 only one hospital provided radiotherapy for the needs of the entire state population.

There were 396 files in our study period of patients with mouth cancer seen at the reference hospital. Table 1 shows the are distribution; lesions predominated in patients over age 60 years (57.8%); the highest rate was in the group over 70 years in both sexes: 45-9% of patients were female and 25.2% were male. The mean age was 63 years in female patients and 61.5 years in males; this diverges from other published results.^12^, ^13^, ^14^, ^15^, ^16^, ^17^

Cancer occurs when cell multiplication processes fail; there are indications that disease progression may vary depending on clinical or pathological features. Thus, the outcome of cancer in elderly patients may be different than that in younger patients.

Young patients with mouth and oropharyngeal epidermoid cancer are more susceptible to the harmful effects of alcohol abuse and smoking; this is probably due to genetic disorders or immune defficienty.^18^,^19^

We were unable to gather complete data on alcohol abuse, smoking and the family history, since files had not been appropriately filled in. These items used to be categorized as optional information; thus, they were absent in about 360 files.

The tongue was the most frequent site (45.71%), followed by the floor of the mouth (18.43%) and lips (9.85%) (Table 2). These results coincide with other published data in that the tongue is the most common site;^13^, ^15^ there was, however, no consensus with the other sites.^13^,^16^

There was a high rate of patients with incomplete basic education (46.56%), not necessarily different from other authors, although these rates in the literature vary depending on the region.^20^ Generally, mouth cancer patients in this study had a low level of formal education, which may reflect the sample, which was taken from a public hospital.^21^

Most of the patients were small farmers (53.9%); these individuals are continuously exposed to sunlight, and often handle carcinogens, both of which may lead to cancer. Nevertheless, some authors argue that occupation is not related with the genesis of mouth cancer,^22^,^23^ and that only 5% of all cancer deaths are truly related with their professional occupations, which therefore poses little risk in general; on the other hand, sunlight is recognized as an important factor in this disease.^24^

Most of the patients were brown-skinned, which diverges from the literature,^13^,^15^ in which white is the most common race. It has been argued that the prevalence of any race is related with the degree of racial mixing in any given region,^20^ which makes such comparisons difficult.

Table 3 shows that the percentage of stage II patients was 57.04%; the percentage of stage III patients was 28.52%. This result agrees with other findings,16 demonstrating that many patients are still diagnosed at advanced stages.

The presence of cases at advanced disease stages may be due to difficulty in accessing medical and dental services, the fear that patients have of discovering themselves with cancer (thus seeking healthcare late), diagnostic difficulties of this disease by dentists and physicians, lack of informative campaigns to sensitize the population about basic and specialized care of the disease at initial stages.^25^

A further explanation for late diagnosis of mouth cancer in the state of Alagoas is that patients are not aware of the signs and symptoms of mouth cancer. Alagoas is not a wealthy state; its economy is agricultural, its HDI is low, most patients do not have even basic education and are of low income, which increases their general susceptibility to diseases.

Staging was not informed in 105 files (26.52%). The consequences of not using such important items in specialized cancer healthcare units include poor follow-up, difficulties in carrying out prospective and retrospective studies with statistical analyses, given the lack of reliable and valid data in the files.

A possible reason for not filling in this information may have been failure or lack of knowledge on the part of health professionals (physicians, dentists and other) about the importance of these data.

There are basically three forms of treatment for mouth cancers: surgery, radiotherapy, and chemotherapy.^12^ Radiotherapy was the most common treatment in our sample, followed by surgery (Table 4); these findings diverge from those in the literature we consulted.^12^ This result reflects the demand and greater ease for undertaking radiotherapy; surgical specialists and infrastructure in this specialty are also lacking in the city. It reflects the difficulties of the Brazilian public health system for high complexity diagnosis and treatment.

## CONCLUSION

Based on our data, we concluded that:

• the epidemiology of mouth cancer in the state of Alagoas shows a higher prevalence in males in the sixth decade of life, on the tongue, in brown colored subjects;
• There was a positive correlation among mouth cancer and some aspects of the HDI-M and living conditions (LCI);
• radiotherapy and surgery were the most frequent types of treatment for mouth cancer in this group;
• patients diagnosed in the initial stage comprised 59.1% of the sample, whereas patients diagnosed at advanced stages comprised 40.9% of the sample;
• healthcare professionals frequently failed to fill in data about patients with mouth cancer (TNM, smoking and drinking alcohol). These data are relevant for providing a more reliable view of this disease.
